# Deep Learning–Assisted Automated Diagnosis of Osteoporosis Based on Computed Tomography Scans: Systematic Review and Meta-Analysis

**DOI:** 10.2196/77155

**Published:** 2025-11-24

**Authors:** Aobo Wang, Ziqian Ma, Tianyi Wang, Ruiyuan Chen, Yu Xi, Qichao Wu, Shuo Yuan, Ning Fan, Peng Du, Lei Zang

**Affiliations:** 1 Department of Orthopedics, Beijing Chaoyang Hospital, Capital Medical University, 5 JingYuan Road, Shijingshan District, Beijing, 100043, China, 86 51718268

**Keywords:** deep learning, artificial intelligence, osteoporosis, computed tomography, meta-analysis

## Abstract

**Background:**

Osteoporosis is a prevalent skeletal disorder characterized by decreased bone mass and increased fracture risk; however, it frequently remains underdiagnosed due to limited health care resources and its asymptomatic progression. Deep learning (DL) provides a promising solution for automated screening using computed tomography (CT) scans, enabling earlier detection and improved management.

**Objective:**

This systematic review and meta-analysis aimed to investigate the diagnostic performance of DL models in diagnosing osteoporosis based on CT scans.

**Methods:**

This study was conducted under the PRISMA (Preferred Reporting Items for Systematic Reviews and Meta-Analyses) guidelines using articles extracted from PubMed, Scopus, Web of Science (Core), and Embase (Ovid). Studies involving adult participants who underwent CT and in which DL was applied for osteoporosis diagnosis were included. The QUADAS-2 (Quality Assessment of Diagnostic Accuracy Studies-2) tool was used to estimate the risk of bias in each study. The confusion matrices from the included studies were extracted to summarize the diagnostic performance of DL models for osteoporosis. Within a bivariate random-effects framework, sensitivity and specificity were jointly synthesized to yield the summary estimates. Heterogeneity was quantified with Higgins I² statistics. Subgroup analyses were performed to explore potential sources of heterogeneity among the included studies.

**Results:**

This review included 24 studies, encompassing CT images from 29,808 participants. All studies used conventional CT scans and used DL-based architectures. Fifteen, 6, and 3 studies were assessed as having a low, uncertain, and high risk of bias, respectively. The meta-analysis included 20 studies. The pooled sensitivity and specificity were 0.88 (95% CI 0.85‐0.91; I^2^=83.69%) and 0.94 (95% CI 0.91‐0.96; I^2^=95.07%) for osteoporosis diagnosis; 0.81 (95% CI 0.76‐0.85; I^2^=82.38%) and 0.92 (95% CI 0.90‐0.94; I^2^=79.05%) for osteopenia identification; and 0.95 (95% CI 0.92‐0.97; I^2^=98.28%) and 0.93 (95% CI 0.91‐0.95; I^2^=94.93%) for normal case identification. The area under the curve of the DL models for identifying osteoporosis, osteopenia, and normal cases was 0.96 (95% CI 0.93‐0.97), 0.94 (95% CI 0.92‐0.96), and 0.98 (95% CI 0.96‐0.99), respectively. Subgroup analyses revealed that models based on DenseNet variants (*P*<.01), multislice input (*P*<.01), 3D architecture (*P*<.01), and CT as the reference standard (*P*<.01) demonstrated superior diagnostic performance.

**Conclusions:**

This study indicated that CT-based DL models achieve promising diagnostic performance for osteoporosis. However, substantial heterogeneity among the included studies, limited external validation, and incomplete end-to-end pipelines constrain the generalizability of the proposed models. Further research is warranted to support their clinical translation and standardized application.

## Introduction

Osteoporosis is a systemic skeletal disorder characterized by low bone mass, deteriorated bone microarchitecture, and increased bone fragility [1]. It affects individuals across diverse ethnic backgrounds, with postmenopausal women being particularly vulnerable. The global prevalence of osteoporosis is estimated at 19.7%, increasing to over 25% among individuals older than 60 years [2], and is expected to increase further with the aging population. In addition, a considerable proportion of individuals are diagnosed with osteopenia, a condition considered a precursor to osteoporosis [3].

Osteoporosis is frequently referred to as a “silent disease” because of its lack of noticeable symptoms in the early stages. However, patients may experience chronic pain and functional impairment as the disease progresses. The most prevalent and severe consequence of osteoporosis is fragility fractures, which significantly reduce patients’ quality of life. In particular, hip fractures are associated with increased mortality. The incidence of osteoporosis-related fractures in China is estimated to reach 4 million by 2030, resulting in over $20 billion in health care costs [4]. Considering the large, affected population, early diagnosis and treatment of osteoporosis are crucial. Interventions such as calcium and vitamin D supplementation and fall prevention strategies have been shown to effectively reduce the risk of fractures [5].

The World Health Organization (WHO) recommends that a T-score of ≤−2.5 measured using dual-energy X-ray absorptiometry (DXA) is indicative of osteoporosis [1]. In recent years, computed tomography (CT) has been recognized as a method that provides more precise and detailed trabecular bone information, making quantitative CT (QCT) a widely accepted diagnostic approach for osteoporosis [6]. However, osteoporosis remains significantly underdiagnosed in clinical practice despite the availability of well-defined diagnostic criteria. This is partly due to limited health care resources, with only 0.46 DXA machines available per million people on average in China [6], and partly due to its asymptomatic nature, with most patients being diagnosed only after a fracture event [7]. Therefore, developing a more accessible and widely applicable screening method for osteoporosis is essential.

The automated analysis of medical images is being actively developed and applied in clinical settings with the rapid advancement of artificial intelligence, particularly deep learning (DL) technologies. As a state-of-the-art approach, DL can directly process raw medical images and leverage large-scale data for training, thereby reducing the reliance on manual feature extraction required in traditional machine learning methods. Moreover, DL has demonstrated superior performance in complex pattern recognition tasks, where subtle imaging features are difficult to predefine [8]. Compared with dedicated osteoporosis screening devices, DL enables the development of scalable screening approaches using existing medical images. For instance, routine chest and abdominal CT scans frequently contain sufficient bone information. DL can emulate the principles of QCT by leveraging vertebral imaging data to enable automated bone mineral density (BMD) assessment, thereby expanding the scope of opportunistic osteoporosis detection. Considering the substantial overlap between patients undergoing CT scans for other medical conditions and those at risk of osteoporosis, this approach may represent a cost-effective strategy for improving osteoporosis management.

However, DL technologies for osteoporosis assessment remain in the developmental and validation phases. Evidence from existing studies must be synthesized to assess the progress in this field and to identify the gaps between model development and clinical application. Therefore, a systematic review and meta-analysis were conducted. This study primarily aimed to evaluate the performance of DL technology in diagnosing osteoporosis and osteopenia based on CT scans and, secondarily, to determine the potential factors affecting the capability of automated diagnosis.

## Methods

### Study Design and Registration

The review was conducted following the PRISMA (Preferred Reporting Items for Systematic Reviews and Meta-Analyses) guidelines and flowchart [9,10] and the PRISMA of Diagnostic Test Accuracy (PRISMA-DTA) checklist [11]. The protocol for this systematic review was registered in the Prospective Register of Systematic Reviews (PROSPERO; CRD42024601713).

### Search Strategy

A comprehensive and exhaustive search was conducted in PubMed, Scopus, Web of Science (Core Collection), and Embase (via Ovid) up to September 28, 2025, to determine relevant articles that used DL techniques for diagnosing osteoporosis based on CT images [12]. Further, the reference lists of the included articles were manually screened to identify additional eligible studies. The following terms were used for the PubMed search: “osteoporosis,” “computed tomography,” “deep learning,” and “neural networks, computer.” Multimedia Appendix 1 provides the details of the search strategy.

### Inclusion and Exclusion Criteria

The inclusion criteria for the studies were (1) full-text articles in peer-reviewed journals; (2) studies involving adult patients who underwent conventional CT scans for routine clinical indications, including chest, abdomen, lumbar spine, or pelvic CT, with or without contrast enhancement; (3) the use of DL methods for osteoporosis detection (classification) or bone density estimation (regression); (4) availability of test dataset information, particularly studies that reported or enabled reconstruction of a 2×2 or 3×3 confusion matrix based on sensitivity, specificity, precision, and recall.

The exclusion criteria were (1) non-English or non–peer-reviewed publications; (2) conference articles, preprints, reviews, letters, guidelines, editorials, or errata; (3) studies with fewer than 30 participants in either the training or test dataset; (4) DL used only applied to image segmentation, while traditional machine learning, radiomics, or HU-to-BMD conversion formulas were used for osteoporosis detection or bone density estimation; (5) studies used specific, nonconventional CT scanning protocols to acquire the input images for the DL model, such as QCT or dual-energy CT protocols (while QCT can serve as the reference standard for osteoporosis diagnosis, the input data for all included DL models were derived from routine CT scans rather than dedicated QCT images).

### Review Process

Two reviewers (ZM and AW) independently performed the initial screening of the titles and abstracts of the included articles to identify potential eligibility after removing duplicates with EndNote (Clarivate). The full texts of the remaining articles were then reviewed, and those not meeting the inclusion criteria were excluded from the study. Any discrepancies were resolved through discussion or adjudicated by a third reviewer (LZ) when necessary. An email was sent to the corresponding authors for the acquisition of the necessary data for studies included in the systematic review but lacking sufficient data for meta-analysis.

### Quality Assessment

The risk of bias and applicability were assessed with the QUADAS-2 (Quality Assessment of Diagnostic Accuracy Studies-2), a tool designed to evaluate the quality of primary diagnostic accuracy studies [13]. The QUADAS-2 criteria were used to examine the risk of bias in 4 domains: Patient Selection, evaluating whether the studies reported general characteristics of the cohorts used for model development and whether the selection of participants was appropriate; Index Test, assessing whether the design and implementation of the models contained any obvious flaws, whether an independent test set was used, and whether the model outputs involved any manual intervention; Reference Standard, examining whether the diagnostic criteria for osteoporosis adhered to internationally recommended guidelines; and Flow and Timing, considering whether the study procedures were appropriate and whether there were excessive delays between the index test and the reference standard. Each domain was assessed in terms of the risk of bias, and the first 3 were assessed in terms of concerns regarding applicability.

### Data Extraction

Data extraction was independently conducted by 2 reviewers (AW and ZM) following the PRISMA-DTA guidelines [11]. Discrepancies were resolved through discussion or adjudication by a third reviewer (LZ). Variables from 4 key aspects were extracted and documented: (1) study design, including the first author, publication year, country of authors, number of participants, participant demographics, testing strategy, and reference standard; (2) characteristics of CT imaging, including the type of CT scans, CT vendor, acquisition parameters, scan region, scan plane, and target vertebrae; (3) details of DL algorithms, including name or architecture of the proposed model, network dimensionality, region of interest (ROI) acquisition method, Dice similarity coefficient of automated segmentation, type of input data, and whether end-to-end processing was performed; (4) diagnostic performance metrics, including confusion matrix (for articles using a 3×3 confusion matrix based on the diagnostic classification criteria for osteoporosis, the matrix was transformed into three 2×2 confusion matrices, each corresponding to a comparison between one category and other categories), sensitivity, specificity, area under the receiver operator characteristic curve, and whether the BMD values were predicted.

The meta-analysis involved both internal and external test results from all eligible studies. Each test result was considered an independent observation for studies reporting multiple test results based on different DL models, datasets, labeling, or data input strategies. A temporal test was considered an internal test because the samples were obtained from the same center and the same CT scanner [14]. Six studies assessed the diagnostic performance of multiple existing models. To prevent these studies from disproportionately affecting the pooled results, only the test results of ResNet and DenseNet for osteoporosis diagnosis were recorded in the literature [15-17], whereas the best-performing 2D or 3D ResNet and DenseNet models were documented in the literature [18], as these architectures are among the most commonly used and effective in medical image detection and classification, owing to their efficient training mechanisms and strong feature representation [19]. Two studies reported model performance on subdistrict datasets from the same center. The relevant results were not included as these do not qualify as standard internal or external tests [20,21].

### Statistical Analysis

The MIDAS and METAN modules in Stata (version 17.0; StataCorp) software were used for statistical analyses [22]. These 2 modules can generate pooled results based on the confusion matrix reported in each included study and perform further statistical analyses. Forest plots were generated to visualize the pooled sensitivity and specificity of DL models in osteoporosis diagnosis. Considering the heterogeneity in study design, model architecture, and reference standards among the included studies, a bivariate random-effects model was used. The overall diagnostic accuracy was assessed through summary receiver operator characteristic curve analysis. Heterogeneity between studies was assessed using the Q test and Higgins I² statistics, with the following classification thresholds: 0%‐40%, 30%‐60%, 50%‐90%, and 75%‐100% indicating negligible, moderate, substantial, and considerable heterogeneity, respectively.

Subsequently, further analyses were conducted to evaluate the diagnostic performance of DL models specifically for osteoporosis. A Fagan nomogram was applied to estimate posttest probabilities, facilitating clinical interpretation of model performance. The likelihood ratio (LR) dot plots were stratified into 4 quadrants according to predefined evidence strength thresholds, guiding the decision-making process for model exclusion or confirmation. The Deeks funnel plot symmetry test was used to assess publication bias. Subgroup analyses were conducted to investigate potential sources of heterogeneity across studies. A *P*<.05 indicated statistical significance.

### Ethical Considerations

Ethical approval and informed consent were not required from the participants considering the nature of the systematic review and meta-analysis.

## Results

### Study Overview

**Figure 1.**
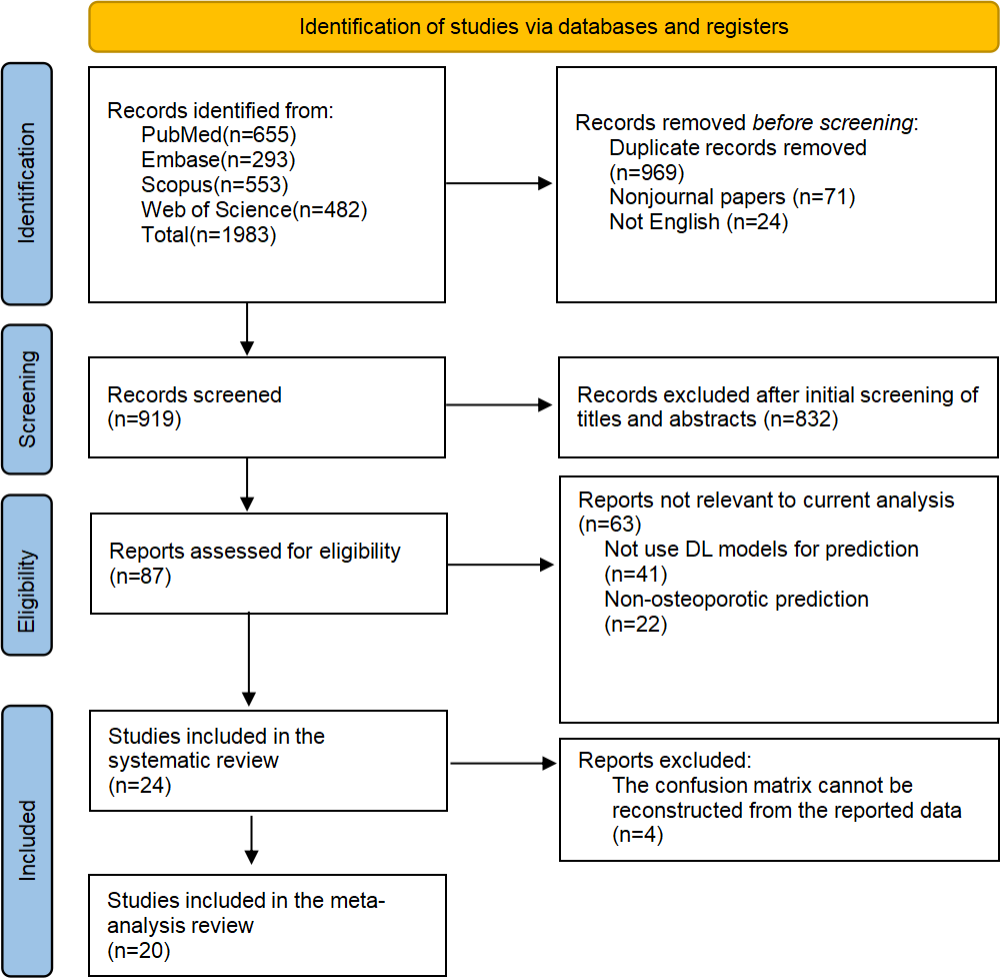
Flowchart depicting the PRISMA (Preferred Reporting Items for Systematic Reviews and Meta-Analyses) search strategy. DL: deep learning.

The literature search was performed according to the protocol outlined in the PRISMA flowchart (Figure 1). The initial search across various databases yielded 1983 studies, including 655, 293, 553, and 482 from PubMed, Embase, Scopus, and Web of Science, respectively. After removing 969 duplicates, 71 nonjournal articles, and 24 non-English publications, 919 publications remained for screening. Of these, 832 were excluded based on titles and abstracts due to lack of relevance. Further, 87 studies were reviewed for full text, of which 24 were included in the systematic review [14-18,20,21,23-39], and 20 were ultimately incorporated in the quantitative meta-analysis [14-16,18,23,25-29]. The other remaining 4 studies were excluded from the meta-analysis because the reported test set sample size and diagnostic parameters were insufficient to reconstruct a complete confusion matrix [24,33,34,39]. Attempts to contact the corresponding authors of the studies did not obtain the necessary data.

The studies included populations from China [14,16-18,20,21,23-26,28,29,32,33,37-39], Japan [27,30], Korea [31,34], the United States [35], Poland [15], and Cyprus [36] geographically. The 24 studies included in the systematic review were published between 2020 and 2025, involving a total of 29,808 participants. Further, 20 studies used single-center data [14-16,18,20,21,23,24,26-29,31-36,38,39], whereas 4 studies used multicenter data [17,25,30,35]. The development, validation, and testing of the DL models included 34,908 samples. In 5 included studies, each participant contributed more than 1 sample, including CT scans from different dates, slices, or vertebral levels [15,31,32,35,36], leading to a total sample size larger than the number of patients. Significant variation in sample sizes was observed, with test sets ranging from 45 [30] to 2867 [23]. The classification forms for osteoporosis diagnosis were summarized into three types: (1) osteoporosis versus osteopenia versus normal cases [14,16,20,21,23-27,29,30,32,33,37-39], (2) osteoporosis versus nonosteoporosis cases [15,17,18,28,34], and (3) low bone mass versus normal cases [31,35,36]. Table 1 shows the detailed information of the included studies.

**Table 1.** Characteristics of the included studies in the systematic review and meta-analysis.

Study	Data source	Population characteristics	Diagnostic classification	Age (years), mean (SD) or median (IQR)	Participants, n (%, female) or %	Test strategy and sample size (n)	Reference standard	Deep learning model networks
Wu et al^a^ [23]	Single-center	Adult, excluded: previous spinal surgery and spinal tumors	Osteoporosis vs osteopenia vs normal cases	54 (IQR 47-61)	7713 (46.50)	Internal: 975; external: 4401	QCT^b^	DenseNet-121
Wang et al [24]	Single-center	Adult, excluded: fractures, metal implants, severe degenerative changes, deformities, and spinal tumors	Osteoporosis vs osteopenia vs normal cases	65 (IQR 57-70)	2274 (44.40)	Internal: 267; temporal: 347	QCT	ResNet-18
Tong ^a^et al [14]	Single-center	Adult, excluded: metal implants, spinal tumors, and abnormal vertebral morphology	Osteoporosis vs osteopenia vs normal cases	62.89 (SD 11.55)	687 (47.20)	Internal: 101	QCT	ResNet
Peng ^a^et al [25]	Multicenter	Adult, excluded: previous spinal surgery, fractures, and tumors	Osteoporosis vs osteopenia vs normal cases	—	1219 (59.80)	Internal: 176; external: 340	QCT	DenseNet
Pan ^a^et al [26]	Single-center	Adult, excluded: metal implants, severe degenerative changes, fractures, and deformity	Osteoporosis vs osteopenia vs normal cases	51 (SD 14.5)	1048 (42.30)	Internal: 418	QCT	ResNet-101
Zhang ^a^et al [16]	Single-center	—^c^	Osteoporosis vs osteopenia vs normal cases	—	—	Internal: 418	QCT	Joint framework
Fang et al^a^ [18]	Single-center	Adult, excluded: severe scoliosis, fractures, and implants	Osteoporosis vs nonosteoporosis cases	—	488 (71.80)	Internal: 96	DXA^d^	Multiple algorithms
Yoshida et al^a^ [27]	Single-center	Adult, excluded: fractures, severe scoliosis, severe spondylosis, prior spinal surgery, and enhanced CT^e^	Osteoporosis vs osteopenia vs normal cases	—	402 (77.40)	Internal: 52	DXA	ResNet-50
Niu ^a^et al [28]	Single-center	Adult, excluded: metal or bone cement implant, secondary osteoporosis, Schmorl’s nodes, severe scoliosis, and vascular calcification.	Osteoporosis vs nonosteoporosis cases	66.03 (SD 9.71)	—	Internal: 100	QCT	DenseNet
Dzierżak and Omiotek^a^ [15]	Single-center	—	Osteoporosis vs nonosteoporosis cases	—	100 (59)	Internal: 100	HU^f^	VGG^g^-16
Fang et al^a^ [29]	Single-center	Adult, excluded: secondary osteoporosis and hyperparathyroidism; fractures and implants	Osteoporosis vs osteopenia vs normal cases	53.8	45.60%	Internal: 398; external: 294	QCT	DenseNet-121
Yasaka et al^a^ [30]	Multicenter	Adult, excluded: previous spinal surgery, severe scoliosis, fractures, and deformity	Osteoporosis vs osteopenia vs normal cases	—	278 (50.80)	Internal: 45; external: 50	DXA	CNN^h^
Kang ^a^et al [31]	Single-center	Adult, the date gap between CT^e^ with a complete L1 axial cut and DXA scan was <1 month	Low bone mass vs normal cases	—	—	Internal: 457	DXA	Residual CNN
Li et al^a^ [32]	Single-center	Patients with cough and epigastric pain as the main symptoms, excluded: malformations, fractures, and abnormal bone metabolism	Osteoporosis vs osteopenia vs normal cases	—	801 (48.80)	Internal: 404	QCT	ResNet
Tang et al [33]	Single-center	Adult, excluded: metastases and compression fractures	Osteoporosis vs osteopenia vs normal cases	—	82%	Internal: 63	DXA	BMDC-Net
Oh et al [34]	Single-center	Adult who underwent routine cancer screening, excluded: implants, and *z* score >3.3 or <−3.3	Osteoporosis vs nonosteoporosis cases	58.86 (SD 12.56)	286 (54.40)	Internal: 98	DXA	DenseNet-169
Tariq et al [35]	Multicenter	Adult, excluded: implants and anatomical variations	Low bone mass vs normal cases	66.9 (SD 9.2)	65.30%	Internal: 1205	DXA	DenseNet-121
Küçükçiloğlu ^a^et al [36]	Single-center	Adult, excluded: severe scoliosis or deformity, spondylarthrosis, inflammatory diseases, tumors, and previous spinal surgery	Low bone mass vs normal cases	—	100 (67)	Internal: 68	DXA	CNN
Zhou ^a^et al [37]	Single-center	Adult, excluded: prior spinal surgery, vertebral fracture, and tumors	Osteoporosis vs osteopenia vs normal cases	47.7	46.1%	Internal: 137	QCT	Resnet-101
Kuo ^a^et al [38]	Single-center	Adult, excluded: prior spinal surgery, vertebral fracture, and tumors	Osteoporosis vs osteopenia vs normal cases	—	507 (66.1)	Internal: 186	DXA	ViT–CNN
Li ^a^et al [20]	Single-center	Adult, excluded: history of previous spinal surgery, severe compression fractures, and tumors	Osteoporosis vs osteopenia vs normal cases	55.11 (SD 13.72)	51.8%	Internal: 245 external: 258	QCT	DenseNet and ResNet
Li ^a^et al [21]	Single-center	Adult, excluded: prior spinal surgery, vertebral fractures, tumors, implants, and BMI >35 kg/m^2^	Osteoporosis vs osteopenia vs normal cases	54.07 (SD 9.90)	987 (59.9)	Internal: 112 external: 137	QCT	DenseNet and ResNet
Zhang et al [39]	Single-center	—	Osteoporosis vs osteopenia vs normal cases	—	—	Internal: 575	QCT	DeepmdQCT
Huang ^a^et al [17]	Multicenter	Inclusion criteria: age ≥50 years, with complete medical records	Osteoporosis vs nonosteoporosis cases	—	1126 (68.7)	External: 545	DXA	Multiple algorithms

a Studies included in meta-analysis (the confusion matrix was either directly provided in the literature or could be reconstructed based on the reported sample size and diagnostic performance metrics, such as sensitivity and specificity).

b QCT: quantitative computed tomography.

c Not available.

d DXA: dual-energy X-ray absorptiometry.

e CT: computed tomography.

f HU: Hounsfield unit.

g VGG: Visual Geometry Group.

h CNN: convolutional neural network.

### Characteristics of CT Images

The types of CT scans used in this systematic review consisted of routine CT [15,16,18,20,24-27,29-33], low-dose CT [14,20,21,23,25,28,37,38], and contrast-enhanced CT [34,35]. The scan regions included chest CT [14,16-18,20,21,23-26,28,31-33,37,38], abdominal CT [25,28-32,34,35], lumbar spine CT [15,20,21,25,29,31,36], and pelvic CT [35]. All used CT images included a complete display of the target vertebral body for osteoporosis diagnosis. Seven studies used sagittal images [20,21,23-25,27,36], 19 used axial images [14-18,20,21,26,28-39], and 3 incorporated coronal images [20,21,35]. Table S1 in Multimedia Appendix 2 provides detailed information on the CT scans.

### DL Model Characteristics

The most commonly used DL architectures in the included studies were ResNet variants [14,16-18,20,21,24,26,27,32,37] and DenseNet variants [17,20,21,23,25,28,29,33-35]. Seven studies used a 3D architecture to predict osteoporosis [14,18,20,21,23,28,31]. Automated segmentation was adopted in 18 studies for ROI localization, in contrast to manual segmentation in 5 studies [15,17,18,27,30]. An end-to-end approach was theoretically feasible in 12 studies among the 24 enrolled studies [14,20,21,23-25,28,29,32,34,35,38]. Osteoporosis was diagnosed directly in 11 studies [14-18,24,26,33,35,36,38] and through BMD-based prediction in 13 studies [20,21,23,25,27-32,34,37,39]. Table S2 in Multimedia Appendix 3 documents further details regarding the DL models.

### Methodological Quality

**Figure 2.**
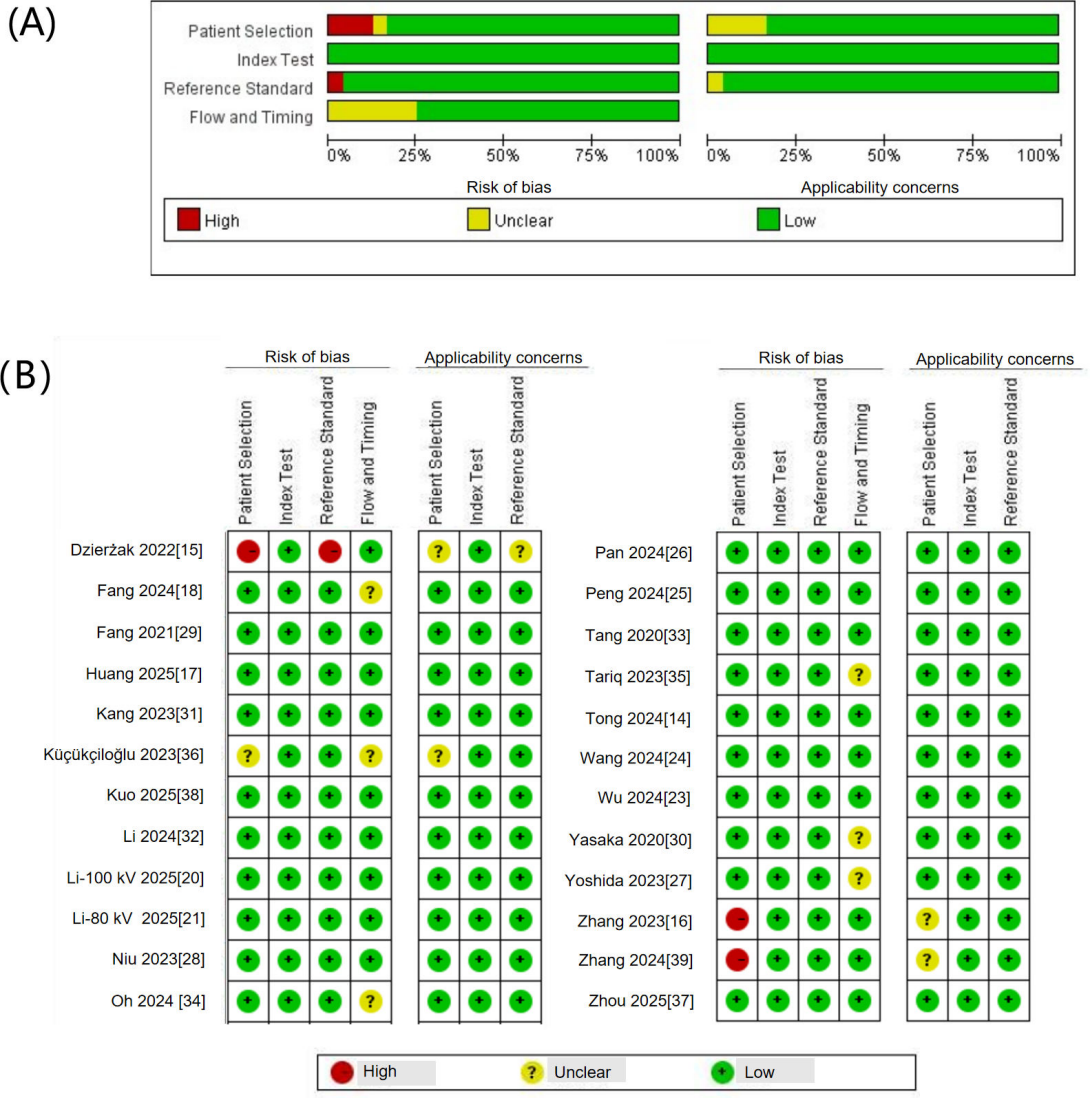
Quality assessment by QUADAS-2 (Quality Assessment of Diagnostic Accuracy Studies-2). (A) The proportion of risk of bias and applicability concerns, and (B) summary of the risk of bias for each study. Green, yellow, and red circles indicate low, unclear, and high risk of bias, respectively.

Figure 2A provides an overview of the quality assessments of the included studies using the QUADAS-2 tool. Figure 2B provides the results of the nuanced analysis. For the risk of bias, 3 studies had a high risk of bias [15,16,39], 6 had an unclear risk of bias [18,27,30,34-36], and 15 had a low risk of bias [14,17,20,21,23-26,28,29,31-33,37,38]. Regarding the applicability, 4 studies had an unclear risk of concern [15,16,36,39]. Regarding the patient selection, 3 of the included studies did not report details of patient selection [15,16,39], and 1 was based on a potentially inappropriate patient population [36], causing a high and unclear bias, respectively. Regarding the index test, all studies enrolled demonstrated a low risk of bias. Regarding the reference standard, both DXA and QCT are recognized tools for osteoporosis assessment [40]. One study adopted HU values as the reference standard, contributing to a high risk of bias [15]. Regarding the flow and timing, 6 studies exhibited an unclear risk of bias due to either an excessive time interval between the index test and the reference standard (>3 months) [27,30,34-36] or insufficient information related to the image input [18].

### Diagnostic Performance of the DL Models

**Figure 3.**
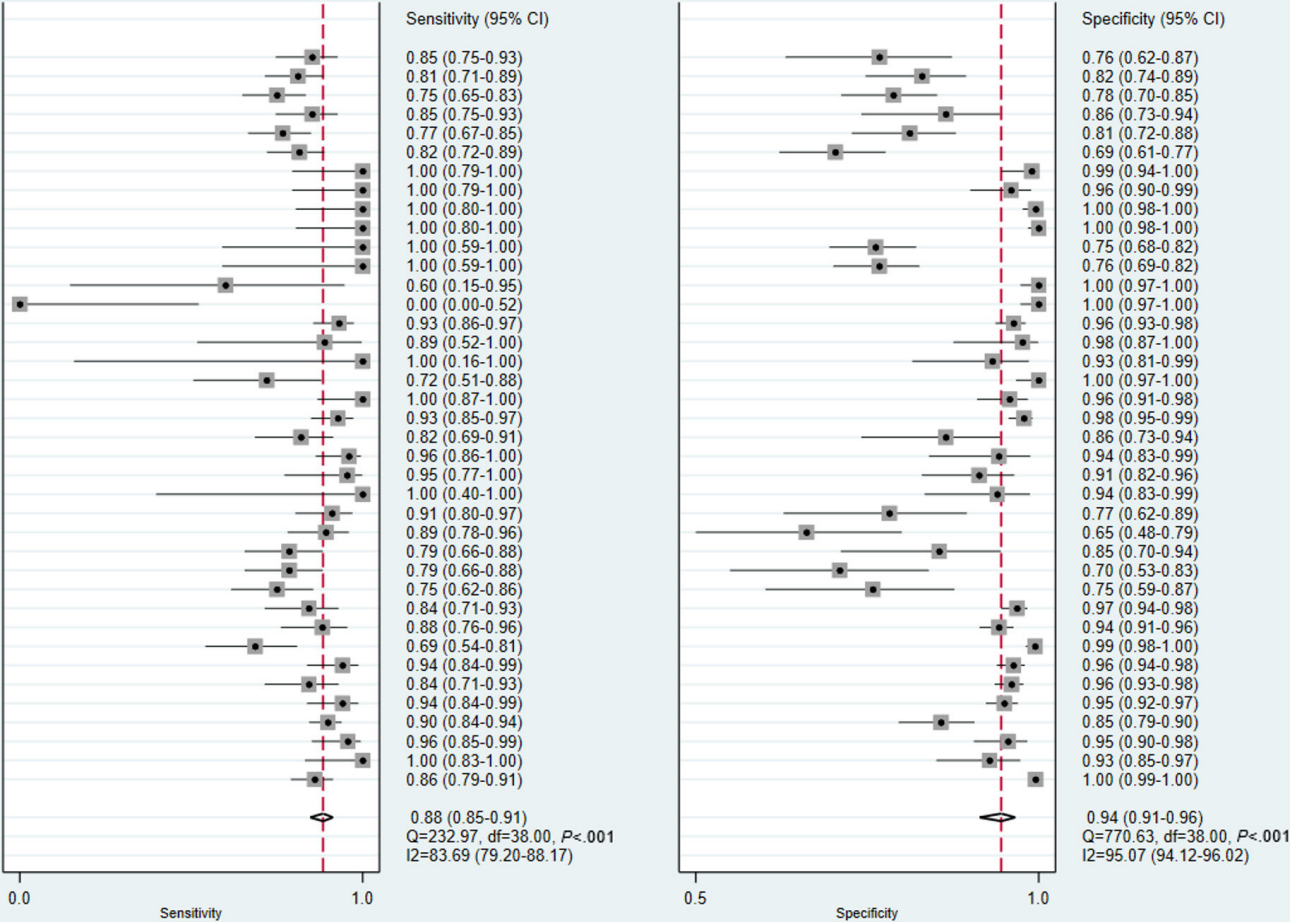
Forest plots in the sensitivity and specificity of deep learning (DL) models in diagnosing osteoporosis.

**Figure 4.**
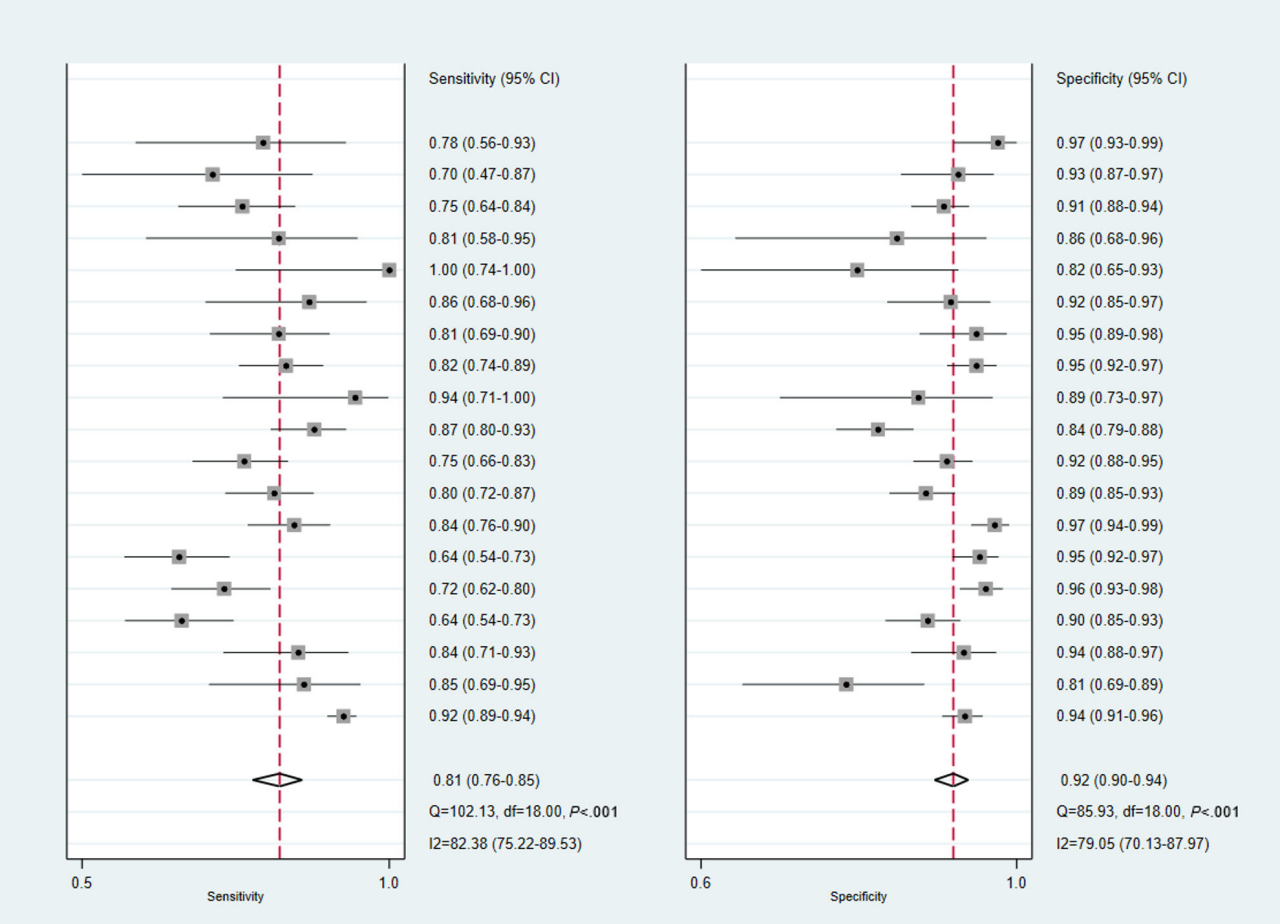
Forest plots in the sensitivity and specificity of deep learning (DL) models in diagnosing osteopenia.

**Figure 5.**
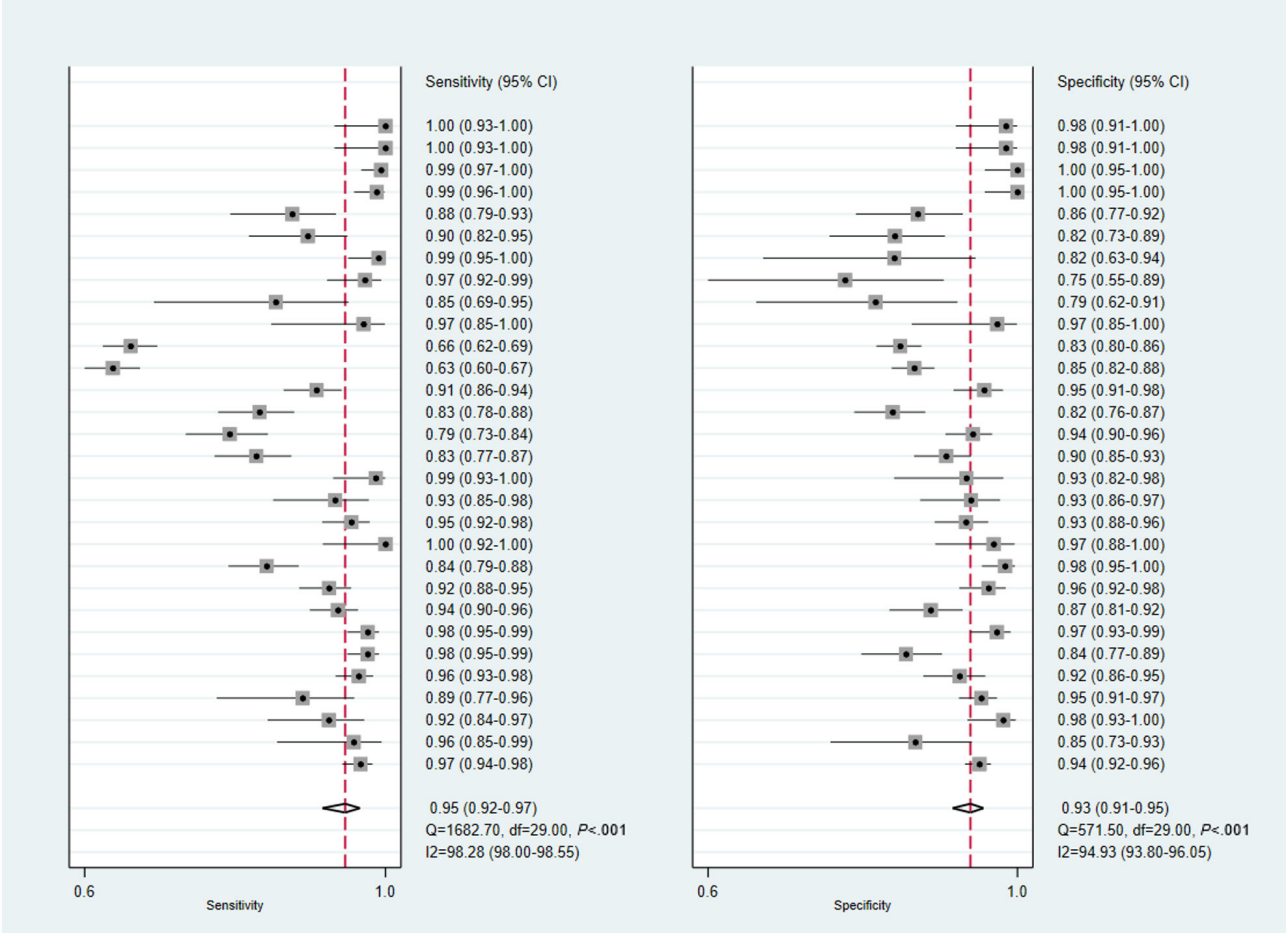
Forest plots in the sensitivity and specificity of deep learning (DL) models in identifying normal cases.

**Figure 6.**
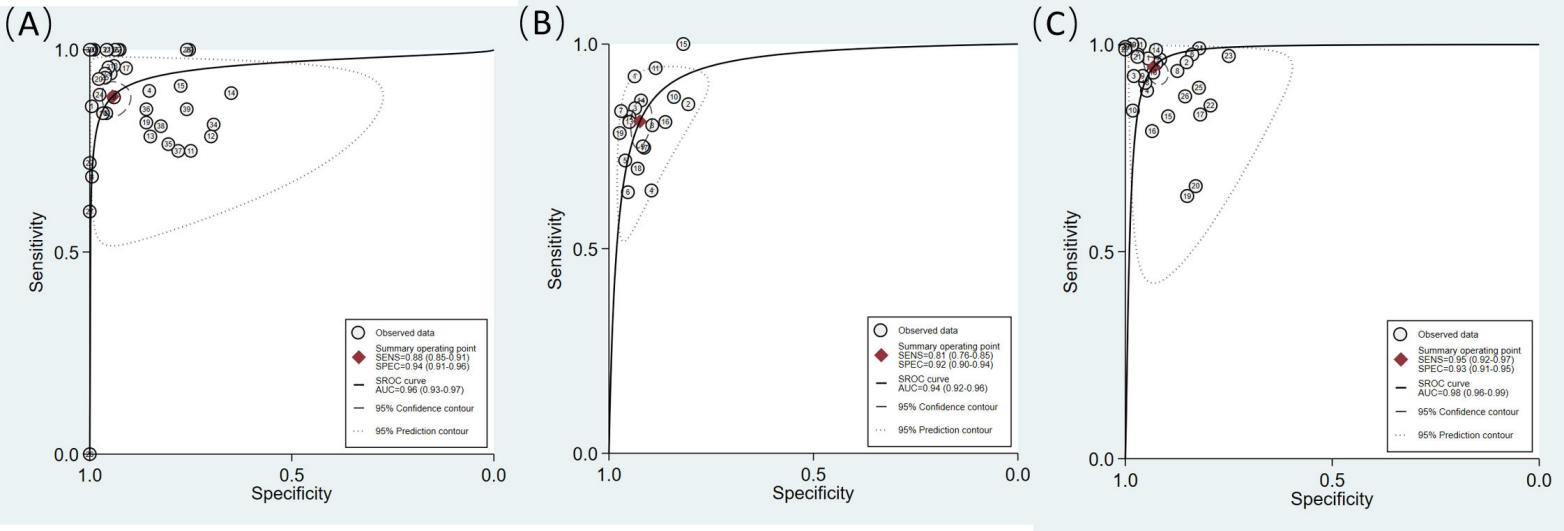
The summary receiver operating characteristic (SROC) curves for deep learning (DL) models in the diagnosis of bone status categories. (A) Osteoporosis, (B) osteopenia, and (C) normal cases. AUC: area under the receiver operator characteristic curve; SENS: sensitivity; SPEC: specificity.

Among the 20 studies included in the meta-analysis, 15 reported diagnostic performance based on multiple model architectures, input data, or test sets [15-18,20,21,25,26,29-31,35-38]. According to the principles described in the “Methods” section, 2‐6 test results from each of these studies were treated as independent observations. Osteoporosis diagnosis involved 39 models. The pooled sensitivity and specificity of osteoporosis diagnosis were 0.88 (95% CI 0.85‐0.91; I^2^=83.69%) and 0.94 (95% CI 0.91‐0.96; I^2^=95.07%), respectively (Figure 3). Osteopenia identification involved 19 models, with pooled sensitivity and specificity of 0.81 (95% CI 0.76‐0.85; I^2^=82.38%) and 0.92 (95% CI 0.90‐0.94; I^2^=79.05%), respectively (Figure 4). Thirty models were included for the identification of normal cases. The pooled sensitivity and specificity for identifying normal cases were 0.95 (95% CI 0.92‐0.97; I^2^=98.28%) and 0.93 (95% CI 0.91‐0.95; I^2^=94.93%), respectively (Figure 5). Considering the substantial heterogeneity across studies, leave-one-out analyses were performed to assess the influence of individual studies on the pooled results. The findings are provided in Figures S1, S2, and S3 in MultimediaAppendices 4^6^. The summary receiver operator characteristic curves indicate that the area under the receiver operator characteristic curve of the DL models for identifying osteoporosis, osteopenia, and normal cases was 0.96 (95% CI 0.93‐0.97), 0.94 (95% CI 0.92‐0.96), and 0.98 (95% CI 0.96‐0.99), respectively (Figure 6).

**Figure 7.**
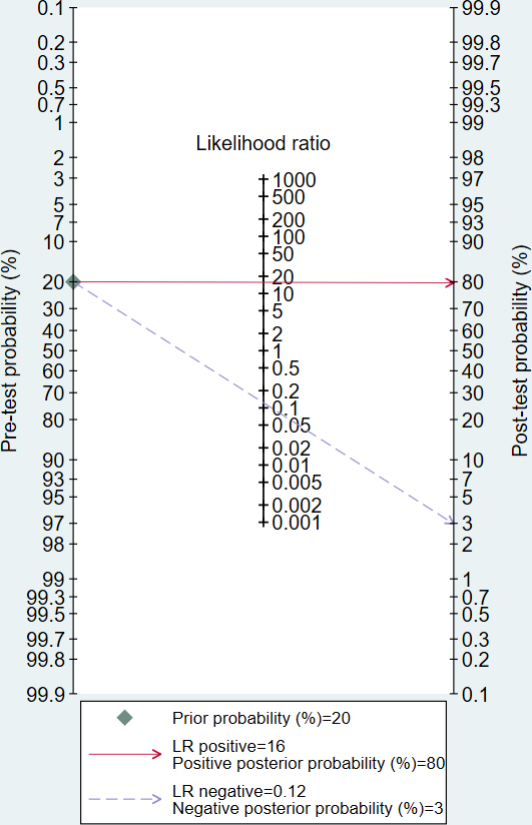
Fagan nomogram of deep learning (DL) models for diagnosing osteoporosis.

According to the prevalence of osteoporosis [2] and the distribution of patients in the included studies, the pretest probability in the Fagan nomogram was set at 20%. At this point, a positive test result of the DL model raises the post-test probability of osteoporosis to 80%, whereas a negative result reduces it to 3% (Figure 7). It should be noted that post-test probabilities depend on the assumed pretest probability. Accordingly, the model’s utility may differ across populations with different baseline prevalence.

**Figure 8.**
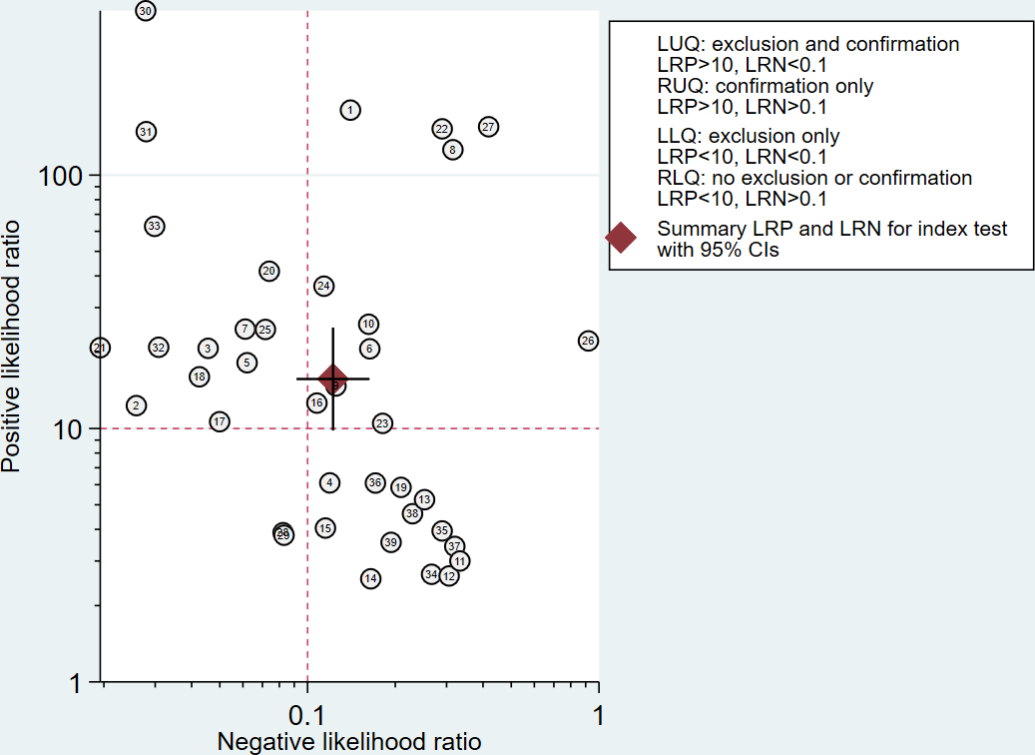
Likelihood ratio (LR) dot plot of deep learning (DL) models. LLQ: lower left quadrant; LRN: negative likelihood ratio; LRP: positive likelihood ratio; LUQ: upper left quadrant; RLQ: lower right quadrant; RUQ: upper right quadrant.

Figure 8 provides the LR scatter plots for osteoporosis diagnosis. Most data points are located in the upper left and upper right quadrants, with the summary LR plot of DL models positioned in the upper right quadrant, indicating the value of DL models in confirming osteoporosis. However, several points fall in the lower right quadrant, demonstrating limited diagnostic use for certain models.

### Publication Bias

The funnel plots and symmetry test were used to evaluate publication bias, indicating whether the results might be skewed due to selective reporting of positive or significant findings (Figure S4 in Multimedia Appendix 7). The funnel plots indicated no publication bias, and the asymmetry test was not significant (*P*=.87).

### Subgroup Analysis

Subgroup analyses were conducted to explore whether specific study characteristics influenced the pooled diagnostic performance and to identify sources of heterogeneity. The included covariates were validation strategy (internal test vs external test), backbone network architecture (ResNet vs DenseNet), scan plane (axial vs sagittal or coronal), image window setting (bone window vs soft tissue window), input data format (single-slice vs multislice), reference standard (CT vs DXA), and model dimensionality (2D vs 3D). The results are provided in Table 2. Models based on DenseNet variants, sagittal and coronal scans, multislice input, CT as the reference standard, and 3D architecture demonstrated superior diagnostic performance. Table S3 in Multimedia Appendix 8 provides detailed sensitivities and specificities for the heterogeneous subgroups.

**Table 2.** Results of subgroup analyses.

Subgroup	Joint model (*P* value)	I²^a^ (%)	LRT^b^ (χ²)^c^
Validation strategy	.08	61	5.15
Backbone network architecture	<.001^d^	96	46.90
Scan plane	<.001^d^	95	41.19
Image window setting	.63	0	0.93
Input data format	<.001^d^	89	18.65
Reference standard	<.001^d^	94	35.71
Model dimensionality	<.001^d^	87	15.19

a I²: I-squared.

b LRT: likelihood ratio test.

c All covariates in the subgroup analyses were binary. Therefore, the degrees of freedom for the LRT test were 2.

d Statistically significant.

## Discussion

### Principal Findings

The rapid development of artificial intelligence, particularly DL, holds the potential to bring significant improvement to health care. DL facilitates opportunistic screening from routine CT scans in osteoporosis diagnosis, enabling early detection and intervention for asymptomatic patients while providing a cost-effective alternative for bone health assessment. However, translating these advancements into clinical practice remains challenging, particularly in terms of generalizability and clinical validation. Therefore, this study aimed to evaluate the diagnostic performance of DL models in osteoporosis detection, emphasizing both progress and limitations. The pooled sensitivity for diagnosing osteoporosis, osteopenia, and normal cases was 0.88, 0.81, and 0.95, respectively, whereas the pooled specificity was 0.94, 0.92, and 0.93, respectively. To our knowledge, this is the first meta-analysis focusing on the use of DL for osteoporosis detection. However, considerable heterogeneity across studies remains, and individual covariates evaluated in the subgroup analysis could not fully account for the observed heterogeneity. In addition, the meta-analysis excluded 4 studies due to insufficient data. Therefore, the results should be interpreted with caution. The pooled estimates should not be interpreted as a single, generalizable performance metric for all DL models, but rather as an overall summary of performance across diverse models, datasets, and clinical settings.

Among studies indexed with “Deep Learning” as a keyword, this systematic review and meta-analysis only included research using DL technology as a classifier. Beyond classification, DL has been used in other components of the automated diagnostic pipeline. A common approach involves using a DL-based localization module to determine ROI, followed by HU- or radiomics-based diagnosing methods [41-45]. HU-based diagnosis estimates BMD using CT density values [46,47]. However, its reliance on a single parameter limits adaptability. Radiomics-based approaches that extract predefined imaging features for prediction face challenges in both feature selection and generalizability. Recent studies indicate that DL-based models may outperform HU- and radiomics-based methods, which may be associated with their capability of automatically learning complex imaging features and leveraging larger datasets [14,18,30]. In addition, DL enables direct feature extraction from raw imaging data, thereby eliminating the reliance on manual feature selection. Compared to previous meta-analyses on HU-based (pooled sensitivity: 63% and specificity: 91%) and radiomics-based (pooled sensitivity: 87% and specificity: 87%) diagnostic methods [48,49], our results indicate a potentially superior performance of DL-based models. However, further direct comparative studies are required to validate this advantage.

Subgroup analysis in terms of model construction revealed that models based on DenseNet demonstrated slightly better performance in osteoporosis diagnosis than those using ResNet. This may be due to DenseNet’s dense connectivity, which improves feature reuse and facilitates stable gradient propagation, enhancing fine-grained image analysis. Studies on viral pneumonia and Alzheimer disease diagnosis reported similar results [50,51], indicating that DenseNet may be better suited for tasks requiring detailed texture and structural assessment. In addition, the use of deep architectures such as ResNet-50 carries an inherent risk of overfitting and suboptimal training performance when applied to small datasets [24]. However, although the 2 architectures showed statistically distinguishable performance, the absolute differences in pooled sensitivity and specificity were minimal, suggesting that the observed significance may not reflect a clinically meaningful superiority. This discrepancy may be attributed to the use of various optimization strategies in most included studies. Future research should conduct more rigorous comparative analyses to identify the optimal model architecture and training strategies for large-scale clinical deployment. Furthermore, it is not surprising that models using multislice input and 3D architecture demonstrated improved diagnostic performance, as these approaches can leverage more comprehensive CT imaging information. Models using sagittal or coronal scans showed superior performance compared with those based on axial scans, possibly because the former provide bone quality information from multiple vertebrae simultaneously. However, the reliability of this finding may be constrained, as studies in the sagittal or coronal subgroup were very limited (n=4).

The results of this study showed no significant difference between the internal and external test performance of DL models. However, this may be attributed to the limited number of eligible external test datasets (n=10), potentially affecting the stability of the pooled estimates. Considering that osteoporosis diagnosis and BMD estimation primarily depend on trabecular bone texture, it is reasonable to assume that factors, such as CT image contrast and clarity, could affect the diagnostic results. Several studies [24,27,39] have demonstrated variations in the performance of DL models when tested on CT images acquired from different vendors and tube voltage settings, which is consistent with our hypothesis.

Models using CT as the reference standard exhibited superior diagnostic performance compared with those using DXA. This is not unexpected, as the outputs tend to be more consistent when the index test and reference standard were derived from the same imaging modality. Although DXA remains the globally recognized gold standard for osteoporosis diagnosis [1], it has known limitations in clinical practice, including the risk of both false negatives and false positives. Lin et al [52] reported that the detection rate of osteoporosis was lower with DXA (73.2%) than with QCT (84.4%) among patients with vertebral fractures—clinically confirmed osteoporosis cases. This discrepancy is caused by DXA being susceptible to osteophytes, degenerative changes, and vascular calcifications [53]. When the reference standard itself is prone to measurement errors, the performance of DL models trained on such data is inherently constrained. Therefore, establishing a more accurate reference standard for osteoporosis diagnosis remains a crucial challenge for future research.

Regarding the diagnostic workflow of DL models, existing studies generally adopt 2 approaches: prediction of BMD values followed by diagnosis, or direct classification. Although these 2 approaches theoretically should not cause differences in diagnostic accuracy, the former may provide practical advantages in clinical applications. First, BMD predictions provide clinicians and patients with a more precise assessment of osteoporosis severity. Second, Peng et al [25] indicated that a primary source of misclassification in DL-based diagnosis involved cases where BMD values are close to classification boundaries. By outputting continuous BMD values instead of discrete diagnostic categories, this approach helps mitigate the effect of misclassification on clinical decision-making.

### Future Directions

Based on the above results and discussion, DL models face practical and ethical challenges in real-world applications beyond diagnostic performance, which should be further addressed in future research and clinical implementation.

First, the considerable heterogeneity observed across the included studies suggests that the performance of current DL models may be influenced by multiple factors, including model architecture and CT acquisition parameters. Therefore, particular caution is warranted when considering their deployment across different scanners and institutions. An alternative strategy for clinical implementation may involve tailoring automated diagnostic models to specific CT vendors and scanner types to ensure diagnostic performance. Future studies on model development should also comprehensively and transparently report details of model design and study data, and conduct more rigorous and extensive cross-institutional and prospective validation to enhance model robustness and generalizability.

DL models encounter practical and ethical challenges in real-world applications beyond diagnostic performance. Only a small proportion of the DL models included in this review were capable of end-to-end processing, as mentioned in the “Results” section. Moreover, Fang et al [29] acknowledged that 14%‐34.5% of cases had invalid CT segmentation results. Similar problems in localization and segmentation further complicate the feasibility of end-to-end automation. These limitations hinder the feasibility of complete automation, requiring human oversight for reviewing and adjusting CT images as warranted. Balancing sensitivity and specificity remains another critical consideration for DL model application. The LR scatter plot indicates that the current DL models predominantly exhibit strong confirmatory capability, posing concerns about their suitability as screening tools. Future research should focus on optimizing the sensitivity of DL models to minimize missed diagnoses while maintaining diagnostic specificity as much as possible.

From an ethical perspective, current DL models primarily serve as diagnostic aids, with physicians integrating DL-generated outputs into their clinical judgment and assuming responsibility for the final diagnosis. However, there is currently no radiological diagnostic standard for osteoporosis that human physicians can directly reference. This indicates that the outputs of DL models may directly constitute the final radiological interpretation, posing ethical concerns regarding accountability and trustworthiness in clinical applications. Therefore, replacing existing osteoporosis diagnostic methods with DL models is not a rational approach in the short term. When DL indicates a risk of osteoporosis, it is necessary to recommend established diagnostic examinations such as DXA and arrange appropriate longitudinal follow-up and treatment. Leveraging the large volume of routine CT examinations for preliminary osteoporosis screening, followed by standardized diagnostic confirmation and appropriate treatment, may improve the overall cost-effectiveness of osteoporosis management.

### Limitations

This study has several limitations. First, although this study used a comprehensive search strategy as thoroughly as possible, the exclusion of non-English and non–peer-reviewed publications may still have resulted in the omission of certain valuable studies. Second, the included studies involved relatively small sample sizes, which may have limited the training effectiveness of DL models. Third, several included studies lacked sufficient reporting of key metrics, making the confusion matrix reconstruction impossible, which could introduce discrepancies between the pooled results and the actual performance. Fourth, the published papers did not always provide complete details of the proposed models, and some of the studies were assessed to have a risk of bias, which limited the interpretability and reproducibility of the results. The complexity of model design and CT parameters also limited our ability to perform further meta-regression analyses. Fifth, multiple test results from individual studies were treated as independent observations in this meta-analysis. Although this approach enabled a more comprehensive inclusion of model performances, it may have introduced potential bias by disproportionately weighting certain study designs. Focusing primarily on the diagnostic performance of ResNet and DenseNet may also represent a source of bias in this study. Finally, no independent validation studies of these models from other institutions were identified in the literature, emphasizing the need for further verification of their generalizability.

### Conclusion

This systematic review and meta-analysis revealed that DL models exhibit promising sensitivity and specificity for osteoporosis diagnosis based on CT images. However, this study also highlights several limitations of existing DL models. First, the included studies showed substantial heterogeneity and lacked robust external validation, which restricts model generalizability; therefore, the pooled results should not be interpreted as a single and universal estimate. Second, some DL models have not achieved a fully end-to-end diagnostic pipeline. Third, the balance between sensitivity and specificity requires further optimization to better align with clinical screening requirements. Despite these limitations, DL techniques hold considerable potential for integration into clinical practice, enabling broader osteoporosis screening and improving cost-effectiveness.

## Supplementary material

10.2196/77155 Multimedia Appendix 1 Full search strings.

10.2196/77155 Multimedia Appendix 2 Characteristics of computed tomography (CT) scans in the included studies.

10.2196/77155 Multimedia Appendix 3 Characteristics of computed tomography (CT) image input and deep learning (DL) algorithms in the included studies.

10.2196/77155 Multimedia Appendix 4 Influence of individual studies on the diagnosis of osteoporosis (leave-one-out analysis).

10.2196/77155 Multimedia Appendix 5 Influence of individual studies on the diagnosis of osteopenia (leave-one-out analysis).

10.2196/77155 Multimedia Appendix 6 Influence of individual studies on the diagnosis of normal cases (leave-one-out analysis).

10.2196/77155 Multimedia Appendix 7 Funnel plot of included studies.

10.2196/77155 Multimedia Appendix 8 Sensitivities and specificities of the heterogeneous subgroups.

10.2196/77155 Checklist 1 PRISMA-DTA (Preferred Reporting Items for Systematic Review and Meta-Analyses of Diagnostic Test Accuracy) checklist.

## Abbreviations

BMD: bone mineral density
CT: computed tomography
DL: deep learning
DXA: dual-energy X-ray absorptiometry
LR: likelihood ratio
PRISMA: Preferred Reporting Items for Systematic Reviews and Meta-Analyses
PRISMA-DTA: Preferred Reporting Items for Systematic Review and Meta-Analyses of Diagnostic Test Accuracy
PROSPERO: Prospective Register of Systematic Reviews
QCT: quantitative CT
QUADAS-2: Quality Assessment of Diagnostic Accuracy Studies-2
ROI: region of interest
WHO: World Health Organization
