# Supplementary material for: Deep Learning–Assisted Automated Diagnosis of Osteoporosis Based on Computed Tomography Scans: Systematic Review and Meta-Analysis
Source: J Med Internet Res. 2025 Nov 24;27:e77155. doi: 10.2196/77155 (PMC12643406; doi:10.2196/77155)
Supplement: Multimedia Appendix 1 [file jmir-v27-e77155-s001.docx]

PubMed: 655

Date searched: September 28, 2025

(((((Convolutional neural network[Text Word]) OR (Deep Learning[Text Word])) OR (Computer Neural Networks[Text Word])) OR (DCNN[Text Word])) OR (Deep transfer learning[Text Word])) OR (Neural network models[Text Word]) OR (Neural networks[Text Word]) OR (Deep neural network[Text Word]) OR (Artificial intelligence[Text Word]) AND ((((Osteoporosis[Text Word]) OR (Osteopenia[Text Word])) OR (Bone mineral density[Text Word])) OR (BMD[Text Word])) OR (Bone density prediction[Text Word]) OR (Osteoporosis screening[Text Word]) AND ((((Computed tomography[Text Word]) OR (Opportunistic CT scan[Text Word])) OR (CT[Text Word])) OR (Quantitative CT[Text Word])) OR (CT-Quantitative[Text Word])

Scopus：553

Date searched: September 28, 2025

( TITLE-ABS-KEY ( "deep learning" OR "convolutional neural network*" OR CNN OR "deep convolutional neural network*" OR "neural network*" OR "transfer learning" OR "artificial intelligence" OR "u-net" OR resnet OR densenet ) ) AND ( TITLE-ABS-KEY ( osteoporosis OR osteopen* OR "bone mineral density" OR "bone densit*" OR BMD OR "fragility fracture*" ) ) AND ( TITLE-ABS-KEY ( "computed tomography" OR CT OR "quantitative computed tomography" OR QCT OR "opportunistic CT" OR opportunistic OR "Hounsfield unit*" OR HU ) ) AND NOT ( TITLE-ABS-KEY ( mice OR mouse OR rat OR murine OR canine OR rabbit OR porcine ) AND NOT TITLE-ABS-KEY ( human OR humans ) )

Web of Science：482

Date searched: September 28, 2025

TS=(

("deep learning" OR "convolutional neural network*" OR CNN OR

"deep convolutional neural network*" OR "neural network*" OR

"transfer learning" OR "artificial intelligence" OR

"u-net" OR resnet OR densenet)

)

AND TS=(

osteoporosis OR osteopen* OR "bone mineral density" OR

"bone densit*" OR BMD OR "fragility fracture*"

)

AND TS=(

"computed tomography" OR CT OR

"quantitative computed tomography" OR QCT OR

"opportunistic CT" OR opportunistic OR

"Hounsfield unit*" OR HU

)

Embase (using the OVID platform): 293

Date searched: September 28, 2025

(exp deep learning/ OR exp neural network/ OR

(deep learning OR convolutional neural network* OR CNN OR

deep convolutional neural network* OR transfer learning OR

neural network* OR artificial intelligence OR

u-net OR resnet OR densenet).ti,ab,kw.)

AND

(exp osteoporosis/ OR osteoporosis.ti,ab,kw. OR osteopen*.ti,ab,kw. OR

exp bone density/ OR "bone mineral density".ti,ab,kw. OR

"bone densit*".ti,ab,kw. OR BMD.ti,ab,kw. OR

"fragility fracture*".ti,ab,kw.)

AND

(exp computed tomography/ OR "computed tomography".ti,ab,kw. OR CT.ti,ab,kw. OR

"quantitative computed tomography".ti,ab,kw. OR QCT.ti,ab,kw. OR

"opportunistic CT".ti,ab,kw. OR opportunistic.ti,ab,kw. OR

"Hounsfield unit*".ti,ab,kw. OR HU.ti,ab,kw.)

NOT (exp animals/ NOT humans/)
