# Supplementary material for: Deep Learning–Assisted Automated Diagnosis of Osteoporosis Based on Computed Tomography Scans: Systematic Review and Meta-Analysis
Source: J Med Internet Res. 2025 Nov 24;27:e77155. doi: 10.2196/77155 (PMC12643406; doi:10.2196/77155)
Supplement: Multimedia Appendix 2 [file jmir-v27-e77155-s002.docx]

**Table S1.** Characteristics of CT scans in the included studies.

| Study | CT scanner | Scan type | Scan region | Scan plane | Tube voltage (kVp) | Tube current (mAs) | CT window | Matrix size |
| --- | --- | --- | --- | --- | --- | --- | --- | --- |
| Wu Y, 2024[23] | Siemens, Philips, GE | LDCT | Chest | Sagittal | 120 | Automatic | Bone | 512×512 |
| Wang S, 2024[24] | Revolution CT (GE) | Routine CT | Chest | Sagittal | 120 | Smart mA | Soft Tissue | 512×512 |
| Tong X, 2024[14] | 256-row CT scanner Revolution CT (GE) | LDCT | Chest | Axial | 80 | Smart mA | Soft Tissue | NA |
| Peng T, 2024[25] | uCT 550, uCT 780 (United Imaging); LightSpeed VCT, Revolution CT(GE) | LDCT and Routine CT | Chest/Abdominal/Lumbar | Sagittal | 120 | Low: 50–70; Routine: automatic | Bone | NA |
| Pan J, 2024[26] | Ingenuity Core 128 CT (Philips) | Routine CT | Chest | Axial | 120 | NA | Soft Tissue | 512×512 |
| Zhang K, 2023[16] | Ingenuity Core 128 CT (Philips) | Routine CT | Chest | Axial | 120 | NA | Soft Tissue | 512×512 |
| Fang K, 2024[18] | NA | Routine CT | Chest | Axial | NA | NA | Bone | NA |
| Yoshida K, 2023[27] | Aquilion ONE, Canon Medical Systems; SOMATOM Force; Siemens; Brilliance iCT; Philips | Routine CT | NA | Sagittal | 120 | NA | Soft Tissue | 256×256 |
| Niu X, 2023[28] | 256-rows detector CT system (GE) | LDCT | Chest/Abdominal | Axial | 120 | SmartmA (10–130) | Soft Tissue | 512×512 |
| Dzierżak R, 2022[15] | 32-row CT scan (GE) | Routine CT | Lumbar | Axial | 120 | NA | Soft Tissue | 512×512 |
| Fang Y, 2021[29] | 128-channel multi-detector CT scanners (uCT 760, United Imaging Healthcare)；Somatom Definition Flash unit (Siemens), Revolution CT scanner (GE) | Routine CT | Abdominal/Lumbar | Axial | 120 | Automatic | Bone | 512×512 |
| Yasaka K, 2020[30] | Aquilion PRIME, Aquilion Precision, Aquilion ONE (Canon Medical Systems) and Discovery CT 750 HD (GE) | Routine CT | Abdominal | Axial | 120 | Automatic | Bone | 512×512 |
| Kang JW, 2023[31] | SOMATOM 128, Definition AS+ scanner (Siemens) | Routine CT | Chest/Abdominal/Lumbar | Axial | 120 | 247 | Bone | NA |
| Li J,2024m[32] | SOMATOM Definition AS (Siemens) | Routine CT | Chest/Abdominal | Axial | 120 | Automatic | Bone | 512×512 |
| Tang C, 2020[33] | Ingenuity CT, Ingenuity Flex, and Brilliance16 | Routine CT | Chest | Axial | 120 | With patient condition | Bone | 512×512 |
| Oh J, 2024[34] | Discovery CT 750HD (GE), Brilliance (Philips), SOMATOM Definition Edge (Siemens) | Contrast-enhanced CT | Abdominal | Axial | 120 or 100 | Automatic | Bone | 392×392 |
| Tariq A,2023[35] | Canon Medical Systems, General Electric Healthcare, Siemens Healthineers | Contrast-enhanced CT | Abdominal/Pelvic | Coronal and axial | 108 (mean) | Automatic | Bone | 512×512 |
| Küçükçiloğlu Y, 2023[36] | 256-detector CT scanner Somatom Definition Flash (Siemens) | Routine CT | Lumbar | Sagittal | NA | NA | Bone | NA |
| Zhou K, 2025[37] | 128-channel multi-detector  CT scanner (uCT 760, United Imaging Healthcare Co.,  Ltd., Shanghai, China) | LDCT | Chest | Axial | 120 | Automatic | Bone | 512×512 |
| Kuo D. P.,2025 [38] | A dual-source CT scanner (SOMATOM Definition Flash; Siemens Healthcare) | LDCT | Chest | Axial | 120 | 200 mA | Bone | 512×512 |
| Li Y-100 kV, 2025 [20] | Six different scanners ((i) Revolution CT, GE Healthcare; (ii) Optima CT620, GE Healthcare; (iii) uCT 790, United Imaging Healthcare; (iv) uCT 820, United Imaging Healthcare; (v) uCT 960, United Imaging Healthcare; (vi) Somatom CT, Siemens Healthineers). | 100 kV CT | Chest | Axial, coronal,  and sagittal | 100 | Smart mA | Bone | 512 × 512 |
| Li Y-80 kV, 2025[21] | Six different scanners (As mentioned above) | 80 kV CT | Chest | Axial, coronal,  and sagittal | 80 | 500 mA (80kV) and Smart mA | Bone | 512 × 512 |
| Zhang K, 2024[39] | The Philips Health Care system from Holland, SOMATOM Force from Siemens | Routine CT | NA | Axial | NA | iPatient  and CARE Dose 4D modulation  technology | Mediastinal window | NA |
| Huang C, 2025[17] | NA | Non-contrast CT | Chest | Axial | NA | NA | Bone | NA |

CT computed tomography. LDCT low-dose chest CT. NA Not applicable
