# Supplementary material for: Deep Learning–Assisted Automated Diagnosis of Osteoporosis Based on Computed Tomography Scans: Systematic Review and Meta-Analysis
Source: J Med Internet Res. 2025 Nov 24;27:e77155. doi: 10.2196/77155 (PMC12643406; doi:10.2196/77155)
Supplement: Multimedia Appendix 3 [file jmir-v27-e77155-s003.docx]

**Table S2.** Characteristics of computed tomography (CT) image input and deep learning (DL) algorithms of included studies.

| Study | Input data type^a^ | Target vertebrae | Utilization of multiple vertebrae information | ROI gain | DSC | 3D model | End-to-end | BMD prediction | R² |
| --- | --- | --- | --- | --- | --- | --- | --- | --- | --- |
| Wu Y, 2024[23] | Volumetric data | T12-L2 | Multi-vertebra Image Input | DL-based | 0.958 | Yes | Yes | Yes | 0.987 |
| Wang S, 2024[24] | Single-slice data | T10-T12 | Multi-vertebra Image Input | DL-based | 0.95 | No | Yes | No | / |
| Tong X, 2024[14] | Volumetric data | T10-12 | Multi-vertebra Image Input | DL-based | 0.96 | Yes | Yes | No | / |
| Peng T, 2024[25] | Volumetric data | NA | Multi-vertebra Image Input | DL-based | NA | No | Yes | Yes | 0.962 and  0.878 |
| Pan J, 2024[26] | Single-slice data | L1-2 | Feature Fusion | DL-based | 0.95 | No | No | No | / |
| Zhang K, 2023[16] | Multi-slice data | L1-2 | Feature Fusion | DL-based | 0.983 | No | No | No | / |
| Fang K, 2024[18] | Single-slice data/Volumetric data | T12 | NA | Manual | NA | Yes/No | No | No | / |
| Yoshida K, 2023[27] | Single-slice data | L1-4 | Prediction Averaging | Manual | NA | No | No | Yes | 0.81 |
| Niu X, 2023[28] | Volumetric data | T12-L2 | Prediction Averaging | DL-based | 0.95 | Yes | Yes | Yes | 0.952 |
| Dzierżak R, 2022[15] | Single-slice data | L1 | NA | Manual | NA | No | No | No | / |
| Fang Y, 2021[29] | Multi-slice data | L1-4 | Prediction Averaging | DL-based | 0.816-0.880 | No | Yes | Yes | 0.992, 0.986, and 0.980 |
| Yasaka K, 2020[30] | Single-slice data | L2-4 | Prediction Averaging | Manual | NA | No | No | Yes | 0.852,0.840 |
| Kang JW, 2023[31] | Single-slice data | L1 | Prediction Averaging | DL-based | NA | No | No | Yes | 0.856 |
| Li J,2024m[32] | Volumetric data | T11-L4 | NA | DL-based |  | Yes | Yes | Yes | 0.95 |
| Tang C, 2020[33] | Single-slice data | L1 | NA | DL-based | 0.904 | No | No | No | / |
| Oh J, 2024[34] | Single-slice data | L1-4 | Prediction Averaging | DL-based | 0.9683 | No | Yes | Yes | 0.714 |
| Tariq A,2023[35] | Single-slice data | L3 | Multi-vertebra Image Input | DL-based | NA | No | Yes | No | / |
| Küçükçiloğlu Y, 2023[36] | Single-slice data | NA | Multi-vertebra Image Input | NA | NA | No | No | No | / |
| Zhou K, 2025[37] | Multi-slice data | L1-L2 | Prediction Averaging | DL-based | 0.974 | No | No | Yes | 0.944 |
| Kuo D. P.,2025 [38] | Multi-slice data | T7-T10 | Voting | Custom algorithm | NA | No | Yes | No | / |
| Li Y-100 kV, 2025 [20] | Volumetric data | T12 to L2 | Prediction Averaging | DL-based | NA | Yes | Yes | Yes | 0.968–0.986 |
| Li Y-80 kV, 2025[21] | Multi-slice data | T12 to L2 | Prediction Averaging | DL-based | NA | Yes | Yes | Yes | 0.990–0.991 |
| Zhang K, 2024[39] | Multi-slice data | L1-L2 | Feature Fusion | DL-based | NA | No | No | Yes | 0.95 |
| Huang C, 2025[17] | Single-slice data | T12 | NA | Manual | NA | No | No | No | / |

^a^ Single-slice data refers to the use of a single CT slice (eg, a mid-sagittal or mid-axial slice). Multi-slice data involve a set of 2D slices, allowing the model to capture more contextual information while maintaining a 2D architecture. Volumetric data consists of a continuous series of slices forming a full 3D CT volume.

^b^ Multi-vertebra image input refers to using images that simultaneously contain multiple vertebrae as model input. Feature fusion denotes the process of integrating features extracted from multiple vertebrae before making predictions. Prediction averaging involves calculating the mean of BMD predictions from multiple vertebrae to produce a final output.

ROI region of interest, DSC dice similarity coefficient, LDCT low-dose computed tomography, DL deep learning, NA, not available
