# Supplementary material for: Deep Learning–Assisted Automated Diagnosis of Osteoporosis Based on Computed Tomography Scans: Systematic Review and Meta-Analysis
Source: J Med Internet Res. 2025 Nov 24;27:e77155. doi: 10.2196/77155 (PMC12643406; doi:10.2196/77155)

**Figure S1.** Influence of individual studies in the diagnosis of osteoporosis (leave-one-out analysis).


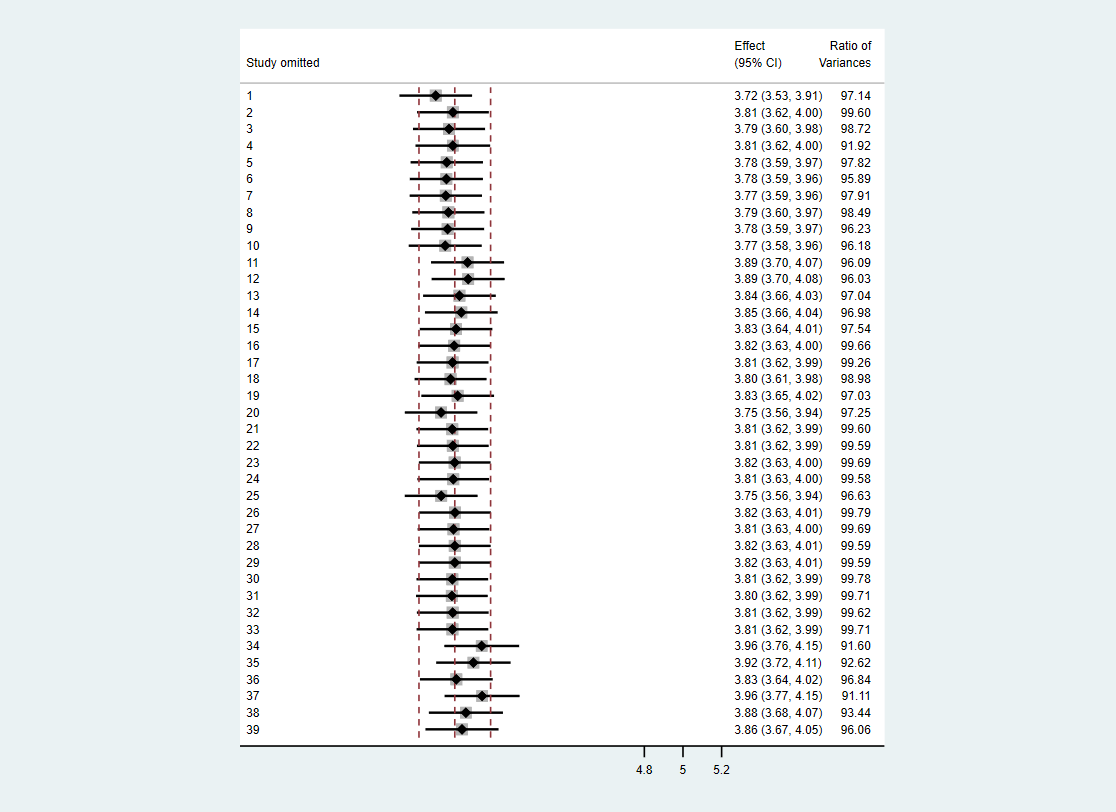

Supplement: Multimedia Appendix 4 [file jmir-v27-e77155-s004.docx]
