# Supplementary material for: Deep Learning–Assisted Automated Diagnosis of Osteoporosis Based on Computed Tomography Scans: Systematic Review and Meta-Analysis
Source: J Med Internet Res. 2025 Nov 24;27:e77155. doi: 10.2196/77155 (PMC12643406; doi:10.2196/77155)
Supplement: Multimedia Appendix 6 [file jmir-v27-e77155-s006.docx]

**Figure S3.** Influence of individual studies in the diagnosis of normal cases (leave-one-out analysis).


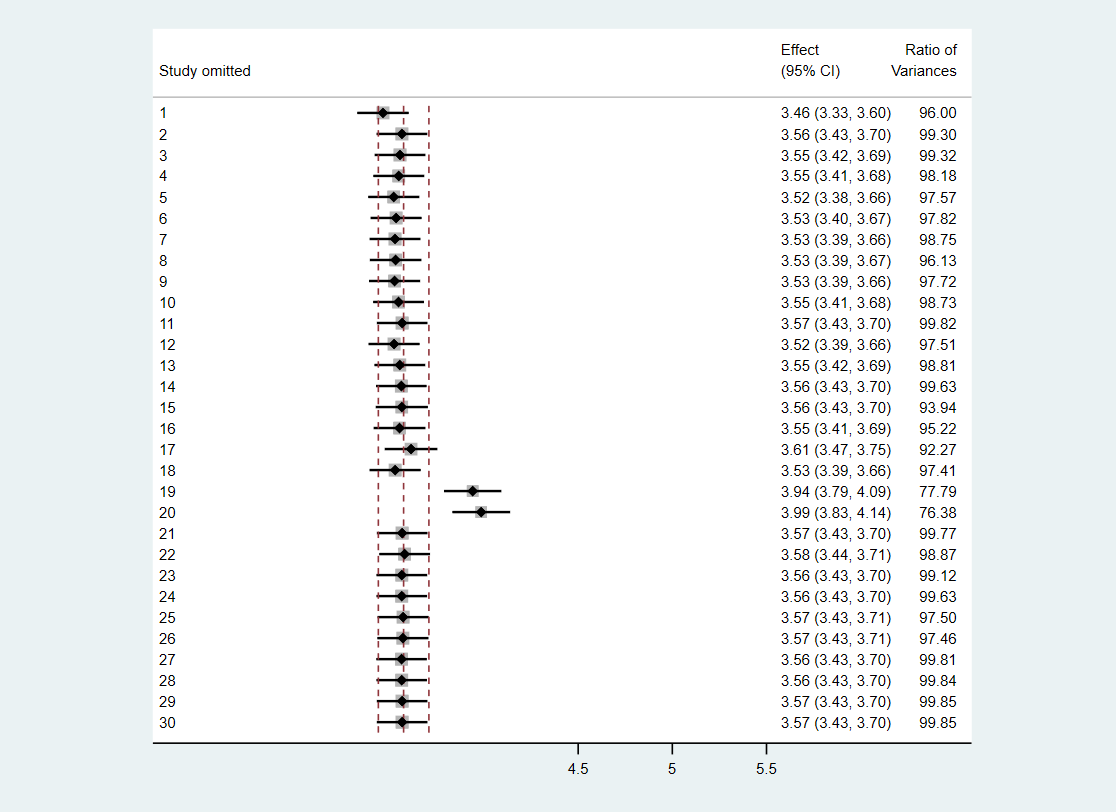


From Figures S1 and S2, it can be observed that models 1# and 4# have a significant impact on the overall variance (ratio of variances <90%). We re-evaluated these two studies. Model 1#, reported by Wu [23], represents the diagnostic performance on the test set, with a relatively large sample size compared to other studies (n=975). Model 4#, reported by Peng [25], represents the diagnostic performance on an external test set. From Figure S3, models 19# and 20# have a significant impact on the overall variance. They are reported by Tariq [35]. We consider the primary reason for this impact is also the relatively large sample size (n=1205). Apart from these factors, we did not identify any other obvious sources of heterogeneity in these studies. Therefore, these studies were retained in the meta-analysis.
