# Supplementary material for: Deep Learning–Assisted Automated Diagnosis of Osteoporosis Based on Computed Tomography Scans: Systematic Review and Meta-Analysis
Source: J Med Internet Res. 2025 Nov 24;27:e77155. doi: 10.2196/77155 (PMC12643406; doi:10.2196/77155)
Supplement: Multimedia Appendix 8 [file jmir-v27-e77155-s008.docx]

**Table S3.** Sensitivities and specificities of the heterogeneous subgroups

| Subgroup | n of models | Sensitivity (95% CI) | Specificity (95% CI) |
| --- | --- | --- | --- |
| Backbone network architecture |  |  |  |
| ResNet | 19 | 0.87 (0.82-0.92) | 0.95 (0.91-0.98) |
| DenseNet | 15 | 0.88 (0.84-0.93) | 0.95 (0.91-0.99) |
| Scan plane |  |  |  |
| Axial | 31 | 0.86 (0.82-0.90) | 0.92 (0.89-0.96) |
| Sagittal/coronal | 4 | 0.91 (0.85-0.98) | 0.96 (0.91-1.00) |
| Input data format |  |  |  |
| Multi-slice | 21 | 0.91 (0.87-0.94) | 0.97 (0.96-0.99) |
| Single-slice | 18 | 0.85 (0.80-0.90) | 0.87 (0.80-0.94) |
| Reference standard |  |  |  |
| DXA | 16 | 0.83 (0.78-0.89) | 0.82 (0.74-0.89) |
| CT | 23 | 0.90 (0.87-0.93) | 0.97 (0.96-0.99) |
| Model dimensionality |  |  |  |
| 2D | 30 | 0.85 (0.82-0.89) | 0.93 (0.89-0.96) |
| 3D | 9 | 0.95 (0.92-9.98) | 0.98 (0.95-1.00) |
